# Heart Failure with Preserved Ejection Fraction and Cardiac Amyloidosis in the Aging Heart

**DOI:** 10.3390/ijms252111519

**Published:** 2024-10-26

**Authors:** Marco Tana, Rachele Piccinini, Livia Moffa, Claudio Tana

**Affiliations:** 1 Internal Medicine and Cardiovascular Ultrasound Unit, Medical Department, SS. Annunziata Hospital, 66100 Chieti, Italy; 2 School of Internal Medicine, Faculty of Medicine, G. D’Annunzio University, 66100 Chieti, Italy; 3 Geriatric Clinic, SS. Annunziata Hospital, 66100 Chieti, Italy

**Keywords:** cardiovascular aging, cardiac amyloidosis, heart failure, preserved ejection fraction, oxidative stress

## Abstract

Heart Failure with Preserved Ejection Fraction (HFpEF) is one of the most frequent causes of heart failure in the world’s population (about 19–55%), and is commonly associated with a high rate of hospitalization (almost 70–80%) and with increased mortality (40–50% in a 5-year timeframe). The elderly are more often affected, with higher rates of hospitalizations than young people, and currently almost 70% of the population aged 65 years old has HFpEF. An increase in cardiomyocyte stiffness, thus resulting in diastolic dysfunction, increased filling pressures and heart failure with preserved ejection fraction are characteristics features of the disease. In addition, among the various causes of HFpEF, cardiac amyloidosis (CA) can provoke diastolic dysfunction and increased wall stiffness directly from intercellular deposition of insoluble proteic substances and their toxic activity. Totally, almost 30 different proteins are able to form deposits, but the most frequently involved are transthyretin and misfolded monoclonal immunoglobulin light chains, which bring to two clinical conditions called transthyretin amyloidosis (ATTR) and light-chain amyloidosis (AL). Although there has been increasing attention on ATTR-CA in recent years, the actual prevalence remains underestimated, especially in people of advanced age, as well as its real impact as a cause of HFpEF, and only data derived from autoptic exams are currently available. Moreover, CA itself often mimics HFpEF, and some conflicting data on the use of predictive scores are described in the literature. The close relationship between HFpEF and CA, especially in older population and the main pathophysiological mechanisms which bond these two conditions are described in this focused review. The need to screen red flags for ATTR-CA in elderly patients with HFpEF is urgently advised, because a prompt recognition of the disease can optimize the approach to the disease with an early therapeutic, life-saving choice.

## 1. Introduction

Heart Failure with Preserved Ejection Fraction (HFpEF) is one of the most frequent causes of heart failure in the world’s population, with a percentage of about 19–55% in various investigation studies) [1,2]. HFpEF is usually associated with a high rate of hospitalization (almost 70–80%) and with high mortality and risk of death (40–50% in a 5-year timeframe) [1,2,3]. Oldest patients are more often affected [4], with higher numbers of hospitalizations than young patients [3]. Currently, almost 70% of the population aged 65 years old has HFpEF [2].

The recent update of the European Society of Cardiology (ESC) has defined HFpEF as the combination of three main elements: symptoms and signs of heart failure, left ventricular ejection fraction (LVEF) ≥ 50% and the evidence of cardiac structural and/or functional abnormalities such as left ventricular (LV) diastolic dysfunction, high LV filling pressures and elevation of NP [5].

Moreover, the disease is often accompanied by several non-cardiac comorbidities such as diabetes mellitus, pulmonary disease, obesity and anemia [6].

An increase in cardiomyocyte stiffness, thus resulting in diastolic dysfunction, increased filling pressures and heart failure with preserved ejection fraction are key pathophysiological elements of this disease [1,2,3,4]. However, among the different causes of HFpEF, cardiac amyloidosis (CA) can cause diastolic dysfunction and increased wall stiffness directly from intercellular deposition of insoluble proteic substances [7,8,9]. In this condition, almost 30 different proteins are able to form deposits [10], but the most frequent involved are transthyretin and misfolded monoclonal immunoglobulin light chains, which bring two clinical conditions called transthyretin amyloidosis (ATTR) and light-chain amyloidosis (AL) [7,8,9].

Two different types of thransthyretin are identified: an acquired wild type, usually associated to ATTRwt-CA, especially in the elderly, and the hereditary misfolded mutated protein, usually linked to ATTRm-CA and to the youngest people [11].

Although there has been an increasing focus on ATTR-CA in recent years, the actual prevalence remains underestimated, especially in the elderly, as well as its real impact as a cause of HFpEF, and only data derived from autoptic exams are currently available [12]. Moreover, CA itself often mimics HFpEF, and some conflicting data on the use of predictive scores are described in the literature [13].

The close relationship between HFpEF and CA, especially in an older population, and the main pathophysiological mechanisms which link these two conditions are described in this focused review. The need to screen red flags for ATTR-CA in elderly patients with HFpEF is urgently advised, because a prompt recognition of the disease can optimize the approach to the disease with an early and appropriate therapeutic, life-saving choice.

## 2. Pathophysiological Features

### 2.1. Heart Failure with Preserved Ejection Fraction (HFpEF)

Hypertrophy of the myocardium, interstitial fibrosis and capillary dysfunction are key pathophysiological elements of HFpEF [14,15].

Pressure and volume overload lead to wall stress that contributes to the immediate release of various substances (such as ATII), which cause the mobilization of inflammatory cells in the myocardium [15]. In this site, an inflammatory cascade is evident and involves numerous proinflammatory cells, release of cytokines, chemokines, resulting in fibrogenesis, and reduction of contractility [16,17,18].

Physiologically, natriuretic peptides (NP) and nitric oxide (NO) activate the conversion of guanylate cyclase (Gc) to cyclic guanosine monophosphate (cGMP) which directly activates protein kinase G (PKG): the latter contributes to the direct phosphorylation of specific proteins, such as titine and troponins, allowing myocardial relaxation and reduction of hypertrophy and fibrosis [18].

In HFpEF, microvascular inflammatory changes and oxidative stress reduce the levels and activity of the NO-cGMP-PKG system, resulting in hypophosphorylation. Specifically, the increase in reactive oxygen species (ROS) directly binds the NO-cGMP-PKG system, inhibiting it, and activates the tissue growth factor (TGF) with fibrogenesis and reduction of elasticity, thus establishing a vicious circle [19,20].

Methionine (Met) and cysteine (Cys) are most commonly involved by oxidative stress with the consequent formation of methionine sulfoxide (MetO) and cys sulfenic, sulfinic and sulfonic acids, respectively [21,22].

However, some authors highlighted that the different cardiac functional and structural alterations may also be triggered by the various comorbidities such as obesity, hypertension, and COPD through a systemic proinflammatory state. The latter causes microvascular endothelial inflammation of the coronary arteries, with consequent reduction of levels and activity of the NO-cGMP-PKG system, titin hypophosphorylation, myocardial hypertrophy and interstitial fibrosis [23].

### 2.2. Cardiac Amyloidosis (CA)

Similarly to HFpEF, pathophysiology of CA is a result of combined triggers that include altered metabolism, inflammation and oxidative damage, changes in intracellular calcium and impaired mithocondrial function [24].

It is well known and largely studied that amyloid infiltration of ventricle intercellular space and valves directly causes increase in wall stiffness, resulting in atria dilatation, increase in pressures and diastolic dysfunction [7,8,9].

However, organ dysfunction secondary to the intercellular deposit of amyloid fibrils is not only generated by tissue mechanical compression, but also by the high toxicity of these substances [25]. The soluble monomers and oligomers such as those present in the AL and ATTR form are able to damage directly myocytes.

Specifically, in AL amyloidosis, light chains are able to provoke directly oxidative stress and can interfere with the redox balance in cardiomyocytes by increasing the reactive oxygen species (ROS) [26,27]. They alter the intracellular exchange of calcium and activate the p38 mitogen-activated protein kinase (MAPK) pathway, resulting in contractile dysfunction and in impaired release of cardiac muscle cells as well as an increase in apoptosis and cell death [28]. Curiously, it has been noted that MAPK signaling increases the transcription of type B natriuretic peptide (BNP) [29] and that high circulating levels of light chains and BNP are related to unfavorable prognostic forms of AL amyloidosis [30].

In ATTR amyloidosis, many authors noted how cardiac dysfunction precedes fibrils deposition, thus confirming the toxic action of pre-fibrillar small soluble monomers and oligomers through various mechanisms, such as increase in ROS, caspases or through a direct interaction with membranes and cholesterol residues, and an increase in apoptosis [14].

### 2.3. The ‘Aging Heart’

Age (especially ≥80 years) is one of the most common determining factors for increasing prevalence of HFpEF [31,32]. Moreover, autopsy examinations have shown how approximately 25% of patients with age >85 years are affected by the acquired variant of transthyretin amyloidosis (ATTRwt) [33], and in 5–10% of them there is a cardiac involvement [34].

Thus, age, HFpEF and CA (especially ATTR-CA) are strictly interconnected: aging is an inevitable process, and, in certain populations, could lead to structural and functional modifications which are the basis for the development of HFpEF and CA.

Advanced age generates not only systemic changes, with direct repercussion on elderly heart, but also indirect modifications through the comorbidities commonly correlated with frailty [35].

Aging is a slow and inevitable process that involves various organs and systems [14]: specifically, different signaling pathways are involved in the cardiac and functional changes in the aging heart that commonly lead to HFpEF [36]. The reduction of the levels of insulin-like growth factor-1 (IGF-1) was strictly linked to a higher risk of developing HF [37] also in older patients without heart disease [38]. Moreover, the elderly are easily prone to develop mitochondrial disturbances and increase of ROS levels [39], thus leading to relaxation and compliance impairment [40].

A low grade, systemic pro-inflammatory state in the absence of any infection is common in the elderly; it is called ‘inflammaging’ and is associated with unfavorable outcomes and prognosis [41]. Many authors noted high levels of TNF-alpha, ROS and inflammatory cells, such as macrophages and monocytes, and of angiotensin II and endothelin. All of these alterations lead to hypertrophy, fibrosis and alteration of relaxation [42].

Interestingly, the aging heart is indirectly affected by the involvement del vascular system: wall stiffening, pulse pressure widening and isolate systolic arterial hypertension are very common finding in older patients [43] and these alterations easily lead to ventricular afterload and decreased cardiac output [44]. A collagen rupture due to oxidative stress, fibrogenesis relative to renin angiotensin aldosterone system (RAAS), insulin resistance and hyperglycemia are the most common pathways involved [45,46].

In addition, the dissociation from actin-myosin structures and extrusion from the myocytes of calcium ions (Ca^2+^) is dysregulated, together with a reduced sensitivity of myofilaments to Ca^2+^ itself, thus resulting in impaired homeostasis of calcium, alteration of cardiac relaxation and diastolic dysfunction [47,48].

Alterations in structure and function of the extracellular matrix (ECM) is responsible for diastolic dysfunction: normally, ECM has the function of a solid proteic structure (collagen, fibronectin, laminin, elastin) of anchoring and aligning cardiomyocytes, and is continuously remodeled by various proteases and matrix metalloproteinases (MMP) to prevent its accumulation.

In addition, transforming growth factor-b (TGF-b) is a profibrotic substance able to up-regulate proteins of ECM and to down-regulate MMPs [49].

In the aging heart, dysregulation of production and remodeling of the ECM, combined with reduced MMPs and higher levels of profibrotic factors, such as TGF-b and connective tissue growth factor (CTGF), lead to an excess of ECM with stiffness of the heart and reduced diastolic function [50].

Deformation of heart shape and structure, such as sigmoid ventricular septum, amyloid infiltrates or brown atrophy, calcific deposits in the valves (especially aortic), together with the increase in epicardial adipocytes and muscle atrophy [51], are the main and characteristic features of aging that favor the onset and maintenance of HFpEF.

Moreover, amyloid fibrils can directly involve the atrial wall [52], in the absence of any ventricular involvement [7]. This phenomenon, called ‘isolated atrial amyloidosis’ (IAA) [7], seems to be related to atrial natriuretic peptide (ANP) accumulation in older patients’ hearts [53]. A study conducted by Yang et al. [54] described the toxic and pro-arrhythmic effects of pre-amyloid oligomeric deposits formed by natriuretic peptides, especially in older patients. The latter, in fact, are more predisposed to the physiological and gradual deposition and accumulation of ANP.

Another study conducted by Röcken et al. [55] highlighted a diffuse amyloid infiltration in 40/245 patients (16.3%) undergoing open heart surgery, and that this abnormal accumulation strictly correlated and predisposed to atrial fibrillation (AF) onset (persistent AF in 38/245, 15.5% patients). Most of amyloid fibrils were immunoreactive for ANP after Congo red staining and immunohistochemistry (40 patients, 16.3%). In this study, amyloid infiltration was strictly related with age (*p* < 0.01).

Moreover, in the last years, growing evidence noted microRNAs as important regulatory elements in the pathogenesis and evolution of cardiovascular diseases, senescence, as well comorbidities that interact negatively with the cardiovascular system (diabetes mellitus, obesity, dyslipidemia and hypertension) [51,56].

Carbonylation of proteins, similarly to HFpEF, with formation of ketones and aldehydes, is another irreversible result of oxidative stress in the elderly [57,58], especially in those over 70–80 years old, and in patients with Parkinson’s or Alzheimer’s Disease [59,60,61].

Interestingly, many authors reported high levels of carbonyled proteins in patients with TTR amyloidosis, with a possible correlation between fibrils and oxidative stress [62,63].

In addition, post-transcriptional modifications of proteins and chaperones that can directly alter and destabilize transthyretin were largely described in older subjects, resulting in their accumulation in the extracellular space of organs, such as the heart [25]. Furthermore, the toxic activity of monomers and oligomers could finally lead to direct damage of the heart in a vicious circle [25].

## 3. Treatment and Preventive Strategies

Adequate sodium intake, reduction of specific nutrients, diet and caloric restriction, alcohol and smoke cessation, regular and daily exercise, reduction of stress and control of lifestyle are all general actions to reduce and slow the aging of the cardiovascular system [14].

In HFpEF patients, only limited and contrasting data regarding the effect of pharmacological therapy on hospitalization and mortality are actually available.

The various studies conducted on diuretics in HFpEF patients observed a clear reduction of symptoms and fluid retention; a meta-analysis of three trials (TOPCAT, HOMAGE, Aldo-CHF), conducted on a total of 984 patients with HFpEF (452 from HOMAGE, 398 from Aldo-DHF, 134 from TOPCAT), treated for 9–12 months with spironolactone and compared to the placebo, showed a reduction of the left atrial volume index (LAVi) by −1.1 pml/m^2^ (*p* = 0.03), interventricular septum (IVS) thickness by −0.2 mm (*p* = 0.01) and an increased left ventricular ejection fraction (LVEF) by 1.7% (*p* < 0.01) [64].

In a randomized, double-blind trial performed by Pitt et al. [65] on 3445 with symptomatic HF and LVEF of 45% or more and treated with spironolactone (15–45 mg daily) or the placebo, the primary outcome of cardiovascular death and aborted cardiac arrest was not different between the two groups (hazard ratio [HR], 0.89; 95% confidence interval [CI], 0.77–1.04; *p* = 0.14). Death and hospitalizations for any causes were similar between the two groups. By contrast, spironolactone reduced hospitalization for HF (HR, 0.83; 95% CI, 0.69 to 0.99, *p* = 0.04).

A recent meta-analysis published on Lancet by Jhund et al. [66] on the RALES and EMPHASIS-HF studies, which enrolled HFrEF subjects treated with spironolactone and eplerenone, respectively, and on the TOPCAT and FINEARTS-HF trials, which enrolled HFmrEF or HFpEF patients treated with spironolactone and finerenone, respectively, showed interesting data on non-steroidal mineralocorticoid receptor antagonists (nsMRAs) and HFmrEF or HFpEF. Specifically, MRAs lowered the risk of cardiovascular death and the rates of HF hospitalization (HR 0.77, 95% CI 0.72–0.83), with benefits from nsMRAs irrespective of EF. Therefore, sMRAs lowered the risk of cardiovascular death or HF hospitalization in HFrEF subjects and nsMRAs reduced this risk in HFmrEF or HFpEF subjects.

Another compound, sacubitril/valsatan, had beneficial effects on decompensations with limited data about the all-cause deaths and cardiovascular mortality [67].

Specifically, the PARAGON-HF trial, a multicenter, randomized and double-blind study that compared sacubitril/valsartan versus valsartan alone in patients with HFpEF showed a reduced rate of hospitalizations in the first subgroup (relative risk [RR] 0.85, 95% CI: 0.72–1.00) without significative benefit on mortality and cardiovascular events [68].

Recently, the use of an antidiabetics drug, the sodium–glucose co-transporter 2 (SGLT2) inhibitors, has shown interesting results on cardiovascular mortality, decompensation and hospitalization rates regardless of the coexistence of diabetes (EMPEROR-Preserved study, DELIVER study) [69,70].

Specifically, in the EMPEROR-Preserved study [69], the group treated with empaglifozin (415/2997, 13.8%) had a lower rate of cardiovascular death (HR, 0.79; 95% CI, 0.69 to 0.90; *p* < 0.001) and of hospitalizations for HF if compared to the placebo group (407 and 541, respectively; HR, 0.73; 95% CI, 0.61 to 0.88; *p* < 0.001), and this was independent of the presence of diabetic disease.

In addition, the DELIVER study [70] randomized 6263 patients with HF and mildly reduced EF (HFmrEF) or HFpEF to dapaglifozin and the placebo. The dapaglifozin group had a lower primary composite outcome of cardiovascular death and worsening HF (*p* = 0.95) with a good safety profile also in older patients.

These compounds inhibit the glucose reabsorption at the proximal renal tubule, thus determining its excretion. Initially conceived as antidiabetic drugs, the SGLT2 inhibitors (SGLT2i) have also proven effective in heart failure, as they reduce the circulating plasma volume of interstitial fluid and increase natriuresis. They also seem to reduce the vascular wall stiffness through direct action on the endothelium [71]. In addition, they have also been shown to reduce myocardial hypertrophy through a sodium–hydrogen exchangers down-regulation, thus resulting in calcium and sodium intracellular exchange [72,73]. Antioxidants effects, together with benefits of oxygen homeostasis, are an interesting additional action of these compounds, leading to reduction of fibrosis, stiffness and of diastolic impairment [74,75].

The recent ESC [76] and AHA/ACC/HFSA guidelines [77] included SGLT2i as strong (Class I) and moderate recommendations for HFpEF, respectively.

In recent years, there has been a growing interest in ATTR-CA and its treatment. In particular, tafamidis, an inhibitor of amyloid tetramer dissociation, has been shown to reduce cardiovascular hospitalizations and all-cause mortality, as well as improve quality of life if compared to placebo, and it was recently recommended in patients with ATTR-CA and good life expectancy [12], especially in the early stages of the disease.

Indeed, a multicenter, double-blind, placebo-controlled, phase 3 trial performed by Maurer et al. [78] showed reduced rates of all-cause mortality (HR, 0.70, 95% CI, 0.56 to 0.96) (*p* < 0.001) and cardiovascular hospitalizations (95% CI, 0.56 to 0.81) in patients treated with tafamidis group if compared to the placebo (n.264 and 177 patients, respectively) with a consensual improvement in quality of life (QoL) and distance for the 6-min walk test (*p* < 0.001).

More recently, another compound, vutrisiran, an inhibitor of transthyretin production, has shown to reduce cardiovascular adverse events and death from any cause if compared to placebo (HR, 0.72; 95% CI, 0.56 to 0.93; *p* = 0.01). Similarly, QoL and a 6 min walk test were improved in the vutrisiran group. Finally, the adverse events rate was similar between the two groups (62% vs. 67%, respectively) [79].

Patients with overlapping HFpeF and CA may also benefit from strategies such as conventional guideline-directed medical therapy (GDMT) used in HF [1]. A study conducted on a total of 2356 ATTR-CA patients (82.3% ATTRwt-CA), of these 220 (11%) treated with SGLT2i, with preserved or reduced EF (45.8% ± 11%), showed an excellent benefit and safety profile. Specifically, SGLT2i treatment lowered the rate of cardiovascular and all-cause mortality (HR: 0.41; 95% CI: 0.24–0.71; *p* < 0.001 and HR: 0.57; 95% CI: 0.37–0.89; *p* = 0.010, respectively), and reduced the rates of HF hospitalization (HR: 0.57; 95% CI: 0.36–0.91; *p* = 0.014), with additional benefits on HF symptoms and renal function, both in reduced and preserved EF [80].

Moreover, CA is usually associated with valvulopaties, especially aortic stenosis (AS), which easily lead to reduced quality of life and worse prognosis: up to 16% of elderly people with severe AS have simultaneous CA and could benefit from transcatheter aortic valve replacement (TAVR).

A recent systematic review and meta-analysis conducted by Cannata et al. [81] on seven observational studies that compared TAVR with conventional medical treatment in CA-AS subjects, and that compared TAVR in CA-AS to AS subjects alone, showed that the risk of death in the TAVR group was lower than the patients drugs-treated (44 vs. 36 patients) (OR 0.23, 95% CI 0.07–0.73, *p* = 0.001) with a similar safety profile between CA-AS and AS alone (OR 1.76, 95% CI 0.91–4.09, *p* = 0.085). However, there was a higher risk of pacemaker implantation in the CA-AS patients treated with TAVR.

In addition to standard treatment, different authors highlighted the possible role of antioxidants, antifibrotics, anti-inflammatory and anti-mitochondrial drugs, soluble guanylate cyclase stimulators, myosin modulators or inhibitors pathways, but their use is not still defined and further studies are needed [82].

## 4. Future Directions

### 4.1. Advancements in Diagnostic and Imaging Techniques

**Figure 1:**
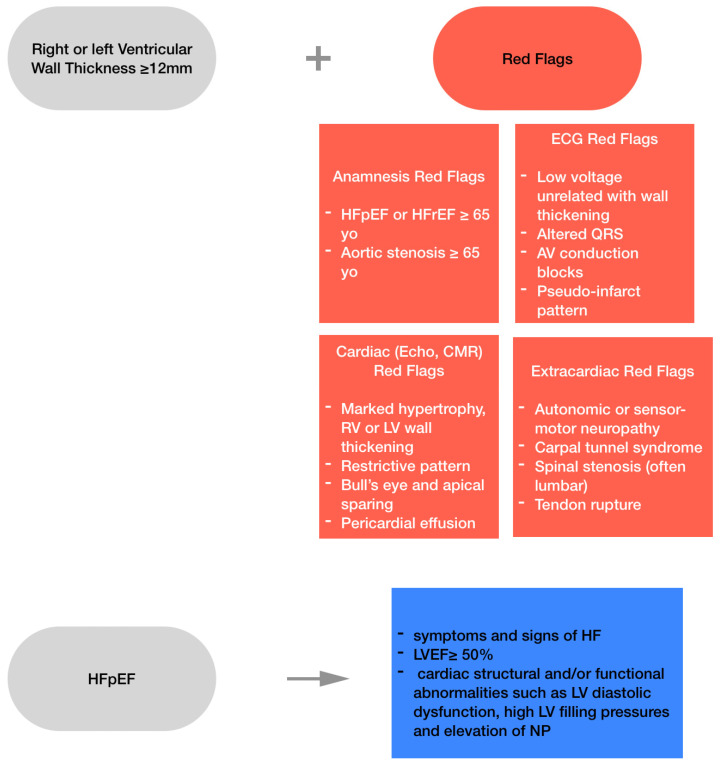
Diagnosis of Cardiac Amyloidosis and Heart Failure with Preserved Ejection Fraction. HFpEF, heart failure with preserved ejection fraction; HFrEF, heart failure with reduced ejection fraction; CMR, cardiac magnetic resonance; ECG, electrocardiogram; yo, years old; AV, atrioventricular; RV, right ventricle; LV, left ventricle; HF, heart failure; LVEF, left ventricular ejection fraction; NP, natriuretic peptide.

Main key elements on diagnosis and future directions are listed in Figure 1 and Table S1, respectively. Recent advances in diagnostic technologies have transformed the detection and management of ATTR-CA and HFpEF. Techniques such as echocardiography, cardiac magnetic resonance imaging (CMRI) and nuclear imaging have greatly enhanced diagnostic accuracy, especially in distinguishing ATTR-CA from other forms of cardiac amyloidosis and heart failure. Nuclear scintigraphy, for example, has become a pivotal tool in non-invasively diagnosing ATTR-CA, reducing the need for invasive endomyocardial biopsies. For HFpEF, cardiac MRI has proven crucial in assessing myocardial fibrosis and diastolic dysfunction, two key pathological features of the condition.

Moreover, new advanced diagnostic methods have been performed: laser-microdissection mass spectroscopy has shown optimal accuracy but been penalized by costs and complex management. Recently, Delrue et al. analyzed 20 transplanted hearts of patients with amyloidosis through the attenuated total reflectance-Fourier transform infrared (ATR-FTIR) spectroscopy, suggesting it as a valid, non-destructive, rapid and economical alternative in the diagnosis of amyloid pathology. However, further studies are needed to validate this methodology [83].

In addition to imaging advances, there is growing interest in developing blood biomarkers for early detection [84]. Biomarkers like NT-proBNP, a marker of cardiac stress, have been widely used in diagnosing heart failure, including HFpEF. Ongoing research is focused on identifying specific biomarkers related to amyloid deposition and myocardial dysfunction, which may allow for earlier interventions before irreversible cardiac damage occurs. These diagnostic improvements enable clinicians to stratify patients based on disease severity and underlying pathology, leading to more targeted and effective treatment strategies.

### 4.2. Therapeutic Developments

Recent breakthroughs in treating ATTR-CA and HFpEF offer new hope to patients. In the case of ATTR-CA, the introduction of transthyretin stabilizers, such as tafamidis, has significantly improved survival rates and quality of life. Additionally, transthyretin hepatic synthesis inhibitors, including patisiran and inotersen, have shown promise in slowing disease progression by targeting the production of misfolded transthyretin proteins. These therapies represent significant advances, although long-term studies are still needed to fully evaluate their safety and efficacy, especially in frail elderly populations.

Treatment options for HFpEF are less well defined due to the heterogeneity of the disease, which stems from its diverse underlying mechanisms. Current strategies focus on managing comorbidities, such as hypertension, atrial fibrillation and diabetes, which often exacerbate HFpEF. Emerging therapies, including antifibrotic agents, anti-inflammatory treatments, and drugs targeting mitochondrial dysfunction, are being actively investigated. For instance, soluble guanylate cyclase stimulators like vericiguat show promise in improving outcomes for HFpEF patients by enhancing myocardial relaxation and reducing fibrosis. Similarly, myosin modulators and inhibitors of key metabolic pathways are being explored as potential treatments.

Antioxidants and anti-inflammatory agents may also play a role in reducing the oxidative stress and chronic inflammation that contribute to HFpEF pathology. However, further studies are necessary to determine their long-term safety, particularly in older patients who are more vulnerable to drug-related side effects.

### 4.3. Challenges in Clinical Management

Despite advancements in diagnosis and treatment, several challenges persist in managing ATTR-CA and HFpEF, particularly in elderly patients who often present with multiple comorbidities. Frailty is a significant concern in this population, complicating treatment decisions and influencing patients’ ability to tolerate medications. A careful balance between therapeutic efficacy and safety must be maintained to prevent exacerbating frailty or causing additional complications.

HFpEF’s heterogeneity presents another challenge in clinical management. Unlike heart failure with reduced ejection fraction (HFrEF), for which well-established treatment guidelines exist, HFpEF lacks a one-size-fits-all approach. The variability in its underlying etiologies requires more personalized treatment strategies tailored to each patient’s specific pathology. Future research should focus on identifying distinct HFpEF phenotypes and developing targeted therapies based on individual patient characteristics.

As for directions in research going forward, research must focus on refining diagnostic tools and treatments for ATTR-CA and HFpEF, especially in aging populations. The development of more precise diagnostic techniques, including genetic screening and advanced biomarker panels, holds promise for earlier detection and better stratification of patients at risk of disease progression. Gene therapy for ATTR-CA is an exciting area of exploration, with the potential to halt disease progression by targeting the root cause—the transthyretin gene. Similarly, for HFpEF, novel drugs targeting fibrosis, inflammation, and energy metabolism may offer more effective treatments tailored to each patient’s unique pathophysiology. Long-term studies are necessary to evaluate how these treatments impact frail and elderly populations, not only in terms of survival but also quality of life and functional independence.

## 5. Conclusions

Aging is a slow and inevitable process, which includes various pathways such as oxidative stress, protein carboxylation and cellular senescence. Many of these mechanisms are shared and constitute a favorable substrate for other pathologies common in the elderly, such as HFpEF and ATTR-CA, in a vicious circle.

As the prevalence of ATTR-CA and HFpEF increases, they will place a growing burden on healthcare systems globally. Early diagnosis and treatment are key to managing this burden, as they can reduce hospitalizations and improve patient outcomes. However, the high cost of new therapies, such as transthyretin stabilizers and gene-silencing drugs, may limit access for many patients, particularly in low-resource settings. Policymakers must consider how to make these treatments more accessible, including through public health initiatives, insurance coverage and pricing strategies that consider the socioeconomic diversity of aging populations. Public awareness campaigns aimed at educating both clinicians and the general population about the signs and symptoms of ATTR-CA and HFpEF could lead to earlier diagnosis and better outcomes. Educational efforts could also reduce misdiagnosis and enable more timely intervention, ultimately lowering healthcare costs associated with the management of these conditions.

In conclusion, while significant progress has been made in diagnosing and treating ATTR-CA and HFpEF, many challenges remain. Early diagnosis, personalized treatment strategies and a focus on improving the quality of life for elderly patients will be essential in managing these diseases effectively. With ongoing research, public health initiatives and policy reforms, the outlook for patients with ATTR-CA and HFpEF is improving, but continued efforts will be necessary to fully capitalize on these advancements.

## Data Availability

Data are contained within this review article.

## Supplementary Materials

The following supporting information can be downloaded at: https://www.mdpi.com/article/10.3390/ijms252111519/s1.
