# Supplementary material for: Heart Failure with Preserved Ejection Fraction and Cardiac Amyloidosis in the Aging Heart
Source: Int J Mol Sci. 2024 Oct 26;25(21):11519. doi: 10.3390/ijms252111519 (PMC11546592; doi:10.3390/ijms252111519)
Supplement: Supplementary file 1 [file ijms-25-11519-s001.zip › ijms-3256507-supplementary.pdf]

Diagnosis		Treatment		
**Imaging**	**Biomarkers**	**Transthyretin stabilizers**	**Transthyretin hepatic synthesis inhibitors**	**TAVR**
Nuclear imaging	NT-proBNP	Tafamidis	Patisiran	Concomitant severe AS
ATR-FTIR spectroscopy	BNP	Vutrisiran	Inotersen	
Laser-microdissection mass spectroscopy	cTnT			
	cTnI			
	hs-cTnT			
	EF			

Table S1. Future directions in patients with Cardiac Amyloidosis and Heart Failure with Preserved Ejection Fraction

ATR-FTIR, Attenuated Total Reflectance-Fourier Transform Infrared; NT-proBNP, N-terminal pro B-type Natriuretic Peptide; BNP, Brain Natriuretic Peptide; cTnT, Cardiac Troponin T; cTnI, Cardiac Troponin I; hs-cTnT, High-Sensitivity Cardiac Troponin T; EF, Ejection Fraction; TAVR, Transcatheter Aortic Valve Replacement; AS, Aortic Stenosis.
